# Molecular Breeding to Improve Salt Tolerance of Rice (*Oryza sativa* L.) in the Red River Delta of Vietnam

**DOI:** 10.1155/2012/949038

**Published:** 2012-12-27

**Authors:** Le Hung Linh, Ta Hong Linh, Tran Dang Xuan, Le Huy Ham, Abdelbagi M. Ismail, Tran Dang Khanh

**Affiliations:** ^1^Agricultural Genetics Institute, Tu Liem, Hanoi, Vietnam; ^2^Graduate School for International Development and Cooperation (IDEC), Hiroshima University, Hiroshima 739-8529, Japan; ^3^International Rice Research Institute, College, Los Baños, Laguna, Philippines

## Abstract

Rice is a stable food in Vietnam and plays a key role in the economy of the country. However, the production and the cultivating areas are adversely affected from the threats of devastation caused by the rise of sea level. Using marker-assisted backcrossing (MABC) to develop a new salt tolerance rice cultivar is one of the feasible methods to cope with these devastating changes. To improve rice salt tolerance in BT7 cultivar, FL478 was used as a donor parent to introgress the *Saltol* QTL conferring salt tolerance into BT7. Three backcrosses were conducted and successfully transferred positive alleles of *Saltol* from FL478 into BT7. The plants numbers IL-30 and IL-32 in BC_3_F_1_ population expected recurrent genome recovery of up to 99.2% and 100%, respectively. These selected lines that carried the *Saltol* alleles were screened in field for their agronomic traits. All improved lines had *Saltol* allele similar to the donor parent FL478, whereas their agronomic performances were the same as the original BT7. We show here the success of improving rice salt tolerance by MABC and the high efficiency of selection in early generations. In the present study, MABC has accelerated the development of superior qualities in the genetic background of BT7.

## 1. Introduction

Salinity is one of the major impediments to enhancing production in rice growing areas worldwide. One-fifth of irrigated arable lands in the world has been reported to be adversely influenced by high soil salinity [1]. As per the report of FAO, 2010 [2], over 800 million ha of worldwide land are severely salt affected and approximately 20% of irrigated areas (about 45 million ha) are estimated to suffer from salinization problems by various degrees. This is more serious since irrigated areas are responsible for one-third of world's food production. In Asia, 21.5 million hectares of land areas are affected by salinity and estimated to cause the loss of up to 50% fertile land by the 21st midcentury [3]. 

 Rice is the most important food crop for over half of the world's population and supplies 20% of daily calories [4]. Rice is a major crop in Vietnam, as the world's second-largest rice exporter after Thailand, together accounting for 50% of the world rice trade. Vast portions of the food producing regions in the country will be inundated by sea water, expected to be at about 19.0% −37.8% of the Mekong River Delta (MRD) and about 1.5% −11.2% of the Red River Delta (RRD). With sea level rise by 1 m, approximately 40,000 km^2^ will be inundated, and salinity intrusion is expected to cover about 71% of the MRD and RRD, together with other coastal regions. Vietnam is formidably dealing with salinity intrusion which is causing adverse influence on 1 million ha, equally with 3% of total Vietnam area [5]. The economic loss by salt intrusion in 2005 was up to 45 million USD, which is equivalent to 1.5% of annual rice productivity in the Mekong Delta [6]. It has a salinity threshold of 3 dS/m, with a 12% reduction in yield per dS/m, beyond this threshold. Therefore, rice yields can be reduced by up to 50% when grown under moderate (6 dS/m) salinity levels [7]. The crop yield reduction in salt soils can be overcome by soil reclamation or by improving salt tolerance in target crops. Therefore, the need for enhancement in salt tolerance in rice is well understood. In the last ten years, a rapid progress has been made towards the development of molecular marker technologies and their application in linkage mapping molecular dissection of the complex agronomical traits and marker-assisted breeding [8]. Rice cultivars grown in saline soil are sensitive at both the vegetative and reproduction stages. However, salinity tolerance at different growth stages seems to be managed by independent genes. *Saltol* is a major quantitative trait locus (QTL) and was identified in the salt-tolerant cultivar Pokkali. Its location was detected on chromosome 1. This QTL confers salinity tolerance at the vegetative stage and explains from 64% to 80% of the phenotypic variance [9]. Several studies reported that this QTL was detected in some other rice varieties [7, 10]. 

 The basis of MABC strategy is to transfer a specific allele at the target locus from a donor line to a recipient line while selecting against donor introgressions across the rest of the genomes [11]. The use of molecular markers, which permit the genetic dissection of the progeny at each generation, increases the speed of the selection process, thus increasing genetic gain per unit time [12]. The main advantages of MABC are (1) efficient foreground selection for the target locus, (2) efficient background selection for the recurrent parent genome, (3) minimization of linkage drag surrounding the locus being introgressed, and (4) rapid breeding of new genotypes with favorable traits. The effectiveness of MABC depends on the availability of closely linked markers and/or franking markers for the target locus, the size of the population, the number of backcrosses, and the position and number of markers for background selection [13]. MABC has previously been used in rice breeding to incorporate the bacterial blight resistance gene *Xa21* [14, 15] and waxy gene [16] into elite cultivars. The availability of the large-effect QTL *Saltol* for salinity tolerance in rice, a theoretical framework for MABC, and the existence of intolerant varieties that are widely accepted by farmers provided an opportunity to develop cultivars that would be suitable for larger areas of submergence-prone rice [17]. Molecular breeding technologies have been widely applied in countries all over the world. It provides powerful tool for development of stress tolerant varieties that can deal with the adverse effects from climate change. However, application of molecular breeding as MABC has just initiated sporadically in Vietnam. Hence, the attempt of this study was to develop a salinity-tolerant version of the widely grown BT7 by applying the MABC method. The improved cultivar may be useful for growing in the soil salinity of the coastal areas of Vietnamese Deltas.

## 2. Materials and Methods

### 2.1. Plant Materials and Crossing Scheme

**Figure 1:**
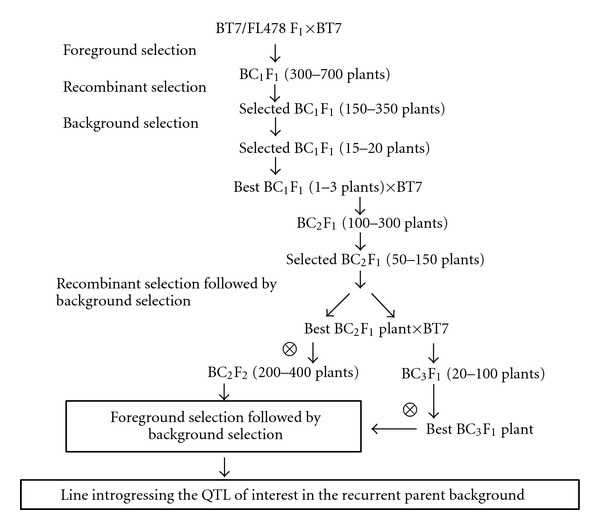
The scheme of applying MABC to improve salt tolerance in BT7 cultivar.

 The scheme for constructing the plant materials used in this study is summarized in Figure 1. A highly-salt tolerant FL478 (IR 66946-3R-178-1-1) was used as the donor of *Saltol* QTLs, whereas BT7 (*O. sativa *spp.* indica*), a popular growing Vietnamese elite cultivar with high quality and popularly grown in the Red River Delta of Vietnam, was used as the recipient parent. A total of 477 SSR markers distributed in the 12 chromosomes including foreground, recombinant, and background markers were screened. For the MABC scheme, BT7 was crossed with FL478 to obtain F1 seeds (Figure 1). F1s were backcrossed with BT7 to obtain a large number of BC_1_F_1_ seeds. In the BC_1_F_1 _generation, individual plants that were heterozygous at the *Saltol *locus were identified reducing the population size for further screening (foreground selection). From the individual plants that were heterozygous for *Saltol*, those that were homozygous for the recipient allele at one marker locus (RM10825) distally franking the *Saltol *locus (i.e., recombinant) were identified. We termed this as “recombinant selection” [18]. Some used markers are shown in detail in Table 1. From these recombinant plants, individuals with the fewest number of markers from the donor genome were selected (background selection). In the second and third BC generations, the same strategy was followed for selection of individual plants with the desired allele combination at the target loci including selection for recombinants between *Saltol *and the nearest proximal marker locus (RM10694) and suitable genomic composition at the nontarget loci and crossed with the recipient parent to develop the next generation. The selected BC2 and BC3 plants were self-pollinated for further analyses. 

### 2.2. Molecular Marker Analysis

 DNA was extracted from juvenile leaves of 2-week-old plants using a modified protocol as described by Zheng et al. (1995) [19]. PCR was performed in 10 *μ*L reactions containing 5–25 ng of DNA template, 1 *μ*L 10X TB buffer (containing 200 mM Tris-HCl pH 8.3, 500 mM KCl, 15 mM MgCl_2_), 1 *μ*L of 1 mM dNTP, 0.50 *μ*L each of 5 *μ*M forward and reverse primers, and 0.25 *μ*L of *Taq *DNA polymerase (4 U/*μ*L) using an MJ Research single or dual 96-well thermal cycler. After initial denaturation for 5 min at 94°C, each cycle comprised 1 min denaturation at 94°C, 1 min annealing at 55°C, and 2 min extension at 72°C with a final extension for 5 min at 72°C at the end of 35 cycles. The PCR products were mixed with bromophenol blue gel loading dye and were analyzed by electrophoresis on 8% polyacrylamide gel using mini vertical polyacrylamide gels for high throughput manual genotyping (CBS Scientific Co. Inc., CA, USA). The gels were stained in 0.5 mg/mL ethidium bromide and were documented using Alpha Imager 1220 (Alpha Innotech, CA, USA). Microsatellite or simple sequence repeat (SSR) markers was used for selection [20].

### 2.3. Foreground and Recombinant Selection

**Figure 2:**
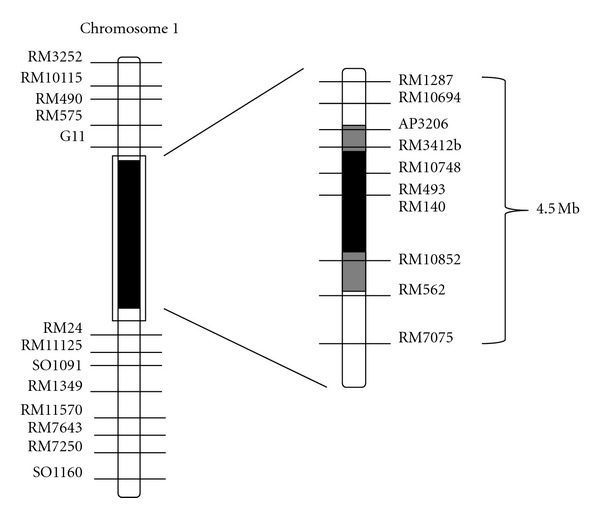
Graphical representation of the regions on chromosome 1 containing *Saltol*. White portions of the bar = homozygous BT7 segment, black regions = homozygous *Saltol *segment, and diagonal slashes = regions where crossing over occurred. Markers polymorphics between B-T7 and FL478 are labeled on both sides of the chromosome. The estimated distances in kb between the SSR markers and their orders are available at http://www.gramene.org/ [21].

 At the initial stages of the experiment, for selection of the *Saltol *locus (foreground), the reported rice microsatellite (RM) markers RM493 and RM3412b, which were found to be tightly linked to *Saltol,* were used for foreground selection. For franking markers used for recombinant selection, about 5 Mb region of the *Saltol *region was targeted. Four polymorphic microsatellite markers (RM1287, RM10694, RM562, and RM7075) were identified for recombinant selection (Table 1, Figure 2). 

### 2.4. Background Selection

**Figure 3:**
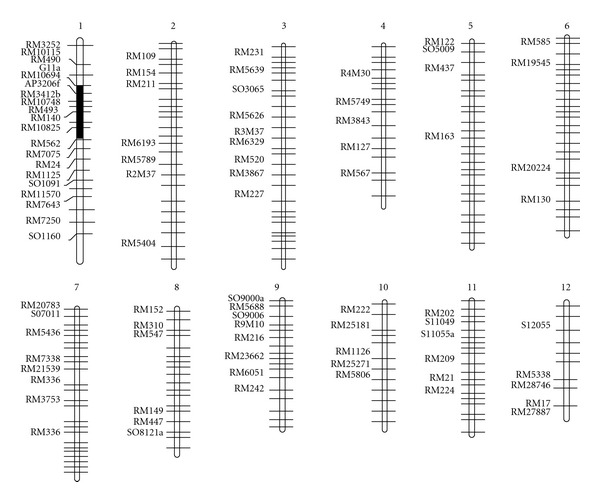
Graphical representation of mapping. Chromosome numbers are at the top of the bars. White portions of the bars are derived from BT7 and dark regions dark region is the linkage between the SSR markers and *Saltol*. Markers polymorphics between BT7 and FL478 are labeled on the left of the chromosomes.

 Microsatellite markers unlinked to *Saltol *covering all the chromosomes including the *Saltol *carrier chromosome 1, that were polymorphic between the two parents, were used for background selection to recover the recipient genome (Figure 3). Based on the polymorphic information, initially evenly spaced microsatellite markers were selected per chromosome. At least four polymorphic microsatellite markers per chromosome were used. The microsatellite markers that revealed fixed (homozygous) alleles at nontarget loci at one generation were not screened at the next BC generation. Only those markers that were not fixed for the recurrent parent allele were analyzed in the following generations. For the selected plants from BC_2_F_1_ and BC_3_F_1_, an additional 84 microsatellite markers were used to check the fixation of the recipient genome. 

### 2.5. Screening for Agronomic Traits

 The BC_3_F_1_ plants with the parents BT7 and FL478 were grown in a field at the Thanh Tri, Hanoi, Vietnam. The plants were laid in a 20 × 15 cm distance and evaluated for 12 traits: (1) days to heading (dth) were evaluated as the number of days from sowing until the panicle headed; (2) plant height (ph) was measured in centimeters from the soil surface to the tip of the tallest panicle (awns excluded); (3) panicle length (pl) was measured in centimeters from the neck to the panicle tip; (4) panicle number (pn) was calculated as the number of panicles per plant; (5) 1,000 seed weight (tsw) was measured in grams as the weight of 1,000 fully filled seeds per plant; (6) primary branch number (pb) was estimated as the number of primary branches per panicle; (7) secondary branch number (sb) was estimated as the number of secondary branch per panicle; (8) seed per panicle (sp) was calculated as the number of fully filled seed per panicle; (9) spikelets per panicle (spp) were calculated as the number of spikelets per panicle.

### 2.6. Statistical Analyses

 The molecular weights of the different alleles were calculated by Alpha Ease Fc 5.0 software. The marker data was analyzed using the software Graphical Genotyper [22]. The homozygous recipient allele, homozygous dominant allele, and heterozygous allele were scored as “A,” “B,” and “H,” respectively. The percentage of markers homozygous for recipient parent (%A) and the percent recipient alleles including heterozygous plants (%R) were calculated. All experimental analyses of the agronomic traits were performed in a completely randomized design at least thrice. Data were analyzed with the use of the Duncan's multiple-range test (*P* < 0.05).

## 3. Results

### 3.1. Foreground and Recombinant Selections

As, the obtained result from screening of 30 SSR markers at the target region on chromosome 1 for polymorphic markers, ten markers showed polymorphics between the parents. Two markers, namely, RM493 and RM3412b, tightly linked to *Saltol,* and four markers RM1287, RM562, RM3252, and RM490 were detected for foreground and recombinant selection, respectively. In each backcross generation (BC_1_F_1_-BC_3_F_1_), the target locus *Saltol *was monitored by markers linked to the *Saltol *genes. Individual BCnF_1_ plants were first selected based on the heterozygous nature of all the target loci at *Saltol *region. Only a few of such selected individuals that had the least donor alleles of the background markers were chosen to be backcrossed with BT7. In advanced backcrosses and selfed generations, polymorphic markers RM493 and RM3412b tightly linked with *Saltol *were used to screen. 

 Four polymorphic markers between BT7 and FL478 at target region were used to screen individual BC_1_F_1_ plants. In conjunction with background selection, the *Saltol* is on chromosome 1 of few selected individuals, including plants number 1, 7, 8 and 26 in BC_2_F_1_, whereas the plants numbers 10, 14, 30, 41, and 359 in BC_3_F_1_ were characterized with two markers for foreground selection (RM493 and RM3412b). When the selected plants of BC_3_F_1_ (plants number 10, 30, 32, and 359) were screened with these two markers, the alleles of markers from RM3412 (12597139 bp) through RM493 (13376867 bp) were of the donor (FL478) type, and the alleles of all the remaining markers from RM1287 (11836436 bp) to RM562 (16232926 bp) onwards were of BT7, indicating that these plants were single recombinants. 

### 3.2. Background Selection

**Figure 4:**
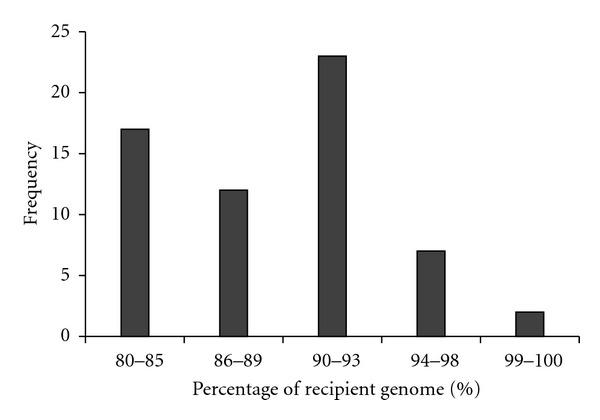
Frequency distribution of the percentage of recurrent parent genome (BT7) in the BC_3_F_1_ population derived from the cross between BT7 and FL478. The vertical axis of each figure represents the relative numbers of BC_3_F_1_ plants.

**Figure 5:**
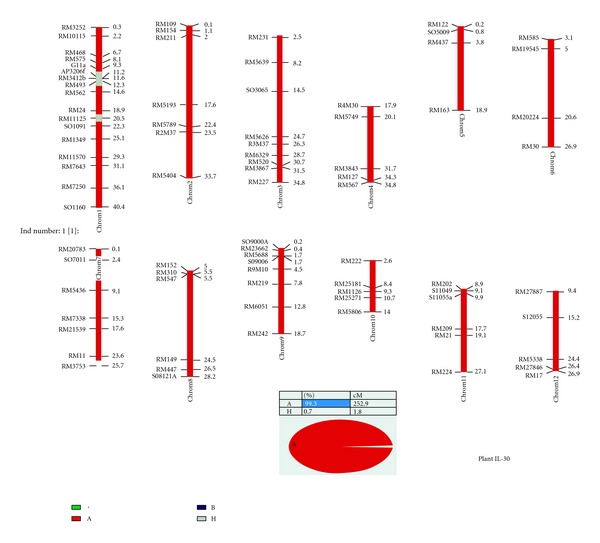
Graphical representation of the plant IL-30 genotype. Chromosome numbers are located at the bottom of the bar. Black portions of the bars are derived from BT7 and slash regions indicated the *Saltol* and FL478 introgressions. Markers are labeled on the right side of the chromosomes.

 A total of 477 SSR markers were screened for polymorphism between BT7 and FL478. Among them, 89 (18.7%) markers showed polymorphisms on 4% polyacrylamide between the parents. The 89 polymorphic markers were used for background selection. The results for polymorphism by SSR marker analysis are diagrammed in Figure 3. In BC_1_F_1_, A total of 30 microsatellite markers were used for background selection in 25 BC_1_F_1_ plants resulting from foreground and recombinant selection (Figures 1 and 2). Based on the foreground and background selection, two selected BC_1_F_1_ plants (nos. 7 and 13) were developed BC_2_F_1_ populations. In the BC_2_F_1_ population, 43 polymorphic markers were used for background selection in 19 BC_2_F_1_ plants resulting from foreground and recombinant selection plants nos. 21 and 41. For plant no. 21 chromosomes 5 and 8 were of complete recipient types. In this experiment, the background analysis of BC_3_F_1_ revealed the recurrent genome recovery of up to 100% at which individual lines were ranging from 81% to 100% as shown in Figure 4. The recurrent genome recovered in the plants no.s IL-30 and IL-32 is expected to be 99.2% and 100%, respectively (Figures 4 and 5).

Table 2 showed the agronomic traits in field screening of the IL to compare with the BT7. In general, there is no significant difference between the morphological traits of IL and BT7. However, the plant height (PH) of IL-30 and IL-32 was 4-5 cm higher than that of BT7. The agronomic traits including day to heading (DTH) and secondary plant number (SP) were similar to those of the recurrent parent, BT7 (Table 2). Moreover, the other traits such as panicle length (PL), panicle number (PN), primary plant number (PN), seed per panicle (SP), spikelets per panicle (spp.), and grain yield, 1.000-grain weight of each the selected lines was almost the same as those of BT7 (Table 2).

## 4. Discussion

 Climate change is causing negative impacts on rice production, which is the most important crop in Vietnam, and its production is mostly confined to the most vulnerable coastal regions. Climate change is severely aggravating the adverse impacts of abiotic stresses on rice production. Most of the rice production lands in coastal areas are already being affected by the rising sea level, increasing the incidences of salinity. However, salt stress problems in field crops can effectively be mitigated through the use of tolerant rice varieties and proper management and mitigation strategies. It is imperative to develop salt tolerance rice cultivars with high yield potential and grain quality using modern tools of biotechnology. However, it is often difficult to incorporate salt tolerance genes into high yielding varieties by conventional breeding methods due to the unexpected linkage drag encountered in the progenies, which affects yield and grain quality characteristics of rice cultivars [23, 24]. Applying molecular breeding has just initiated in some recent years. Lang et al. [25] applied marker-assisted selection (MAS) to improve salt tolerance in OMCS2000 rice cultivar, a widely grown cultivar in North Vietnam. Current efforts in various institutions in Vietnam are directed towards marker-assisted backcrossing strategy to introgress the favourable alleles for salinity tolerance QTLs into elite rice line which could considerably minimize breeding time as well as make arduous screening unnecessary [25, 26].

 It is also challenging to achieve a definite goal of salt tolerance using conventional breeding strategies when the target gene is linked with an unfavorable dominant gene [27]. Nevertheless, since markers have been found as linked to some the specific traits of interest and used as the tools of biotechnology, it is plausible to transfer valuable genes of salt tolerance stresses in rice without linkage drag [28]. In this study, BT7 was selected as the recipient parent because it is good quality rice and always gives high profit for milled rice.

 Our study focuses on combining the useful agronomic traits of BT7 with *Saltol* QTL/gen, which attached salt tolerance in backcross breeding lines by conversion to the recurrent parent genotype using molecular genotyping with SSR markers. We successfully transferred the *Saltol* from donor line FL478 into BT7. The *Saltol* gene was identified in an introgression line, highly salt-tolerant FL478 (IR 66946-3R-178-1-1), which inherited the gene from the Pokkali [29].

Here, we used the MABC breeding method to transfer the *Saltol* gene into a popular cultivar by phenotype and genotype selection. Using SSR markers (RM493 and RM3412b) the *Saltol* gene ensured efficient foreground selection. The codominant nature of SSR markers could be very useful in addition to gene-based markers for the introgression of the *Saltol* locus into a wide range of recipient elite cultivars. The selfed progenies or recombinant homozygote plants in the target region were selected from 300 to 478 plants for each backcrossing generation with foreground selection. Our results demonstrate that a major salt tolerance gene (*Saltol*) from the donor parent FL478 was successfully transferred into the BT7 genetic background and expressed similar phenotypic characteristics when compared with BT7. 

## 5. Conclusions

 We have developed a salt tolerance of BT7 variety by using marker-assisted backcross, which was controlled by a major *Saltol* QTL. The recovery of the recurrent parent genome by molecular genotyping and selection could increase the efficiency of the MABC strategy, and this was achievable in a short span of time. This study could have a good impact on rice breeding, and it is applicable for the introduction of important agronomic traits into the genomes of popular rice cultivars.

## Figures and Tables

**Table 1:** Details of markers for foreground and recombinant selection.

Markers	Mb	Forward primer	Reverse primer	Motif	No. of repeats	SSR start	SSR end
RM10694	11.0	TTTCCCTGGTTTCAAGCTTACG	AGTACGGTACCTTGATGGTAGAAAGG	AC	18	10969040	10969075
AP3206f	11.2	GCAAGAATTAATCCATGTGAAAGA	AGTGCAGGATCTGCCATGA	—	—	—	—
RM3412B	11.6	TGATGGATCTCTGAGGTGTAAAGAGC	TGCACTAATCTTTCTGCCACAGC	—	—	—	—
RM10748	11.8	CATCGGTGACCACCTTCTCC	CCTGTCATCTATCTCCCTCAAGC	AG	14	11758005	11758032
RM493	12.3	GTACGTAAACGCGGAAGGTGACG	CGACGTACGAGATGCCGATCC	AAG	9	12264091	12264117
RM140	12.3	CTTGCACAAGAGATGATGATGAGC	CATGCTGAGAAATAGTACGCTTGG	AG	12	12284725	12284748
RM10825	13.3	GGACACAAGTCCATGATCCTATCC	CTTTCCTTTCCATCCTTGTTGC	AAG	10	13306166	13306195
RM562	14.6	GGAAAGGAAGAATCAGACACAGAGC	GTACCGTTCCTTTCGTCACTTCC	AAG	13	14610402	14610446

**Table 2:** Performance of principal agronomic traits and salt tolerance of the plants numbers IL-30 and IL 32, which were selected as the most promising lines.

Cultivar/breeding line	Agronomic traits									
	*Saltol* present	DTH (d)	PH (cm)	PL (cm)	PN	PB	SB	SP	SPP	TWG (g)
BT7	—	109^a^	107.5^a^	21.0^a^	6.3^a^	8.3^a^	3.5^a^	127^a^	137.6^a^	18.5^a^
FL478	*Saltol *	110^a^	101.2^b^	22.8^b^	7.8^b^	7.6^b^	3.4^a^	106^b^	125.6^b^	28.7^b^
IL-30	*Saltol *	110^a^	110.3^c^	21.2^a^	6.5^a^	8.1^a^	3.5^a^	130^a^	140.1^a^	18.7^a^
IL-32	*Saltol *	110^a^	106.5^a^	21.1^a^	6.5^a^	8.2^a^	3.5^a^	129^a^	139.7^a^	18.8^a^
LSD_(0.05)_		0.27	0.38	0.52	0.08	0.10	0.09	0.61	0.56	0.41

Means with the same letter in a column are not significantly different at *P* < 0.05. Abbreviations present agronomical traits which were presented in Section 2.

## Abbreviation

PCR: Polymerase chain reaction
SSR: Simple sequence repeat marker
MABC: Marker-assisted backcrossing
MAS: Marker-assisted selection
QTL: Quantitative trait loci.
